# Capabilities of computerized decision support systems supporting the nursing process in hospital settings: a scoping review

**DOI:** 10.1186/s12912-025-03272-w

**Published:** 2025-07-01

**Authors:** Cynthia Abi Khalil, Antoine Saab, Jihane Rahme, Jacques Bouaud, Brigitte Seroussi

**Affiliations:** 1 Nursing Administration, Lebanese Hospital Geitaoui–UMC, Beirut, Lebanon; 2 Sorbonne Université, Université Sorbonne Paris Nord, INSERM, LIMICS, Paris, F-75006 France; 3 Quality and Safety Department, Lebanese Hospital Geitaoui–UMC, Beirut, Lebanon; 4 AP-HP, Hôpital Tenon, Paris, France

**Keywords:** Clinical decision support systems, Nursing process, Hospital settings, Scoping review

## Abstract

**Background:**

The implementation of the Nursing process (NP) is a complex and cognitively demanding task that can be enhanced by using clinical decision support systems (CDSSs). While some CDSSs assist in the management of patients with specific patient conditions, nursing process clinical decision support systems (NP-CDSSs) are designed to support nursing decisions through all five sequential NP steps: assessment, nursing diagnosis (NDs), planning, intervention, and evaluation. Although an internationally agreed standard for NP-CDSS development (ICS4NP-CDSSs) was published in 2016, outlining recommended design and functional features for NP-CDSSs, current NP-CDSSs show limited compliance with these guidelines.

**Methods:**

A PubMed search was conducted to identify NP-CDSSs used in hospital settings and published between January 2013 and February 2024. The objective was to assess how well these systems adhered to ICS4NP-CDSSs including coverage of all NP steps, use of standardized nursing languages (SNLs), and compliance with recommended technical features. The search also examined whether NP-CDSSs had been implemented and evaluated and whether any outcome related to system impact or any nurses’ feedback on barriers and facilitators influencing NP-CDSS implementation was documented.

**Results:**

Of the 986 papers retrieved, 35 related to 21 systems met the eligibility criteria. Only seven systems addressed all NP steps, with the evaluation step remaining underdeveloped. Seventeen systems provided NDs based on entered characteristics or risk factors, 15 included automatic linkages between NDs and interventions, but only 10 included linkages to outcomes at the evaluation step. Most retrieved systems used NANDA-I/NANDA, NIC, NOC as SNL. Only four systems reached the post-implementation evaluation phase; none reported patient outcomes analysis, while four collected nursing outcomes. Barriers during implementation included a lack of trust and perceived usefulness to improve daily workflows, limited validation, and missing technical features.

**Conclusions:**

NP-CDSS development is still in its infancy. This review identifies a gap between ICS4NP-CDSS best-practice recommendations and their implementation in current NP-CDSSs, which still lack features like full coverage of NP steps and necessary linkages between them. To enhance NP-CDSSs, strategies should focus on reducing documentation burden, providing comprehensive education and support for nurses, and demonstrating the impact of NP-CDSSs through outcome studies.

**Supplementary Information:**

The online version contains supplementary material available at 10.1186/s12912-025-03272-w.

## Background

Nurses play a pivotal role in healthcare quality and safety through their constant presence at the bedside and continuous interactions with the entire healthcare team. They are key contributors to safety checks, timely coordination, and communication about patient conditions or deterioration [1]. They ensure that patients receive high-quality care, even amid nursing shortages and fast-paced, error-prone health care systems, where information and technology are constantly evolving [2, 3]. This complexity is further exacerbated by the fact that patient harm related to hospital care remains a leading cause of morbidity and mortality [4, 5], causing a high burden on healthcare systems such as extended hospital stays, increased readmission rates, and increased costs [6–8]. Conversely, despite efforts to improve nursing outcomes such as fall prevention and pressure ulcer reduction, recent reports indicate stagnation in these areas [9], alongside a decline in nursing care quality and an increase in missed nursing care [10–14]. These challenges were further exacerbated during the COVID-19 pandemic [15, 16], underscoring the urgent need for innovative solutions to enhance nursing decision-making and outcomes.

In response, artificial intelligence (AI) offers a transformative opportunity to enhance healthcare delivery, particularly through clinical decision support systems (CDSSs) [17]. CDSSs may be defined as computerized systems “providing clinicians with computer-generated clinical knowledge and patient-related information, which is intelligently filtered and presented at appropriate times to enhance patient care” [18]. As direct providers of much of in-hospital care, nurses make multiple decisions that can be supported by CDSSs [3]. Numerous studies have described the success of CDSSs in improving specific nursing clinical practices, such as patient triage in emergency settings, telehealth, prevention of pressure ulcers and falls, administration of anticoagulant drugs, and malnutrition management [19–22]. However, most CDSSs are designed to focus on a single clinical condition [3, 23, 24], whereas hospitalized patients often present with multiple, complex health issues. This single-target approach contrasts with the holistic nature of nursing care, which requires a comprehensive view of a patient’s overall health.

Effective nursing decision-making relies on integrating relevant data to identify Nursing Diagnoses (NDs) and develop individualized care plans through the Nursing Process (NP) [25]. As defined by the American Nurses Association, the Nursing Process is a scientific problem-solving method at the core of standard nursing practice. It guides nurses in delivering holistic, patient-centered care [26], ensuring high-quality care and improving both patient and organizational outcomes, particularly when Standardized Nursing Languages (SNLs) are used [27, 28]. SNLs are structured linguistic systems in which nursing concepts are systematically defined, hierarchically organized, and explicitly related, enhancing the visibility of nursing contributions to patient care, and facilitating patient responses and experiences in health and illness [29]. The American Nurses Association has recognized 12 SNLs, among which the North American Nursing Diagnosis Association International (NANDA-I) is the most widely used [26]. Other notable SNLs include the International Classification for Nursing Practice (ICNP) developed by the International Council of Nurses [30], as well as the Nursing Interventions Classification (NIC) and the Nursing Outcomes Classification (NOC) [31], both of which are frequently used in conjunction with NANDA-I nursing diagnoses. Additionally, the Clinical Care Classification (CCC) provides another standardized framework for nursing documentation and practice [32, 33].

The Nursing Process consists of a cycle of five sequential steps, assessment, nursing diagnosis, planning, intervention, and evaluation [34, 35] as described in Table 1.

**Table 1:** Description of the five nursing process steps

Assessment	Collection of subjective and objective data (vital signs, patient/family interviews, physical exam) necessary to identify health problems/risks relative to the patient.
Nursing diagnosis (ND)	A clinical judgment that focuses on the patient’s *human* response to a health condition, as opposed to a medical diagnosis, which identifies the *physiological* condition/disease behind the health condition. For example, while a medical diagnosis of stroke provides insight about the patient’s pathology, NDs such as *impaired verbal communication, risk of fall, interrupted family processes, and chronic pain,* offer a comprehensive understanding of how the stroke affects the patient’s overall quality of life and that of their family.
Planning	The formulation of nursing goals and outcomes, the planning of interventions, and the documentation of care plans that serve as a structured guide to provide a course of directions for personalized care. A nursing outcome is defined as a measurable behavior or perception exhibited by an individual, a family, a group, or a community, in response to a nursing intervention.
Intervention/implementation	The actual carrying out of nursing interventions with the needed resources outlined in the care plan.
Evaluation of outcomes	Ensuring that desired outcomes have been met. The care plan may be adapted based on new assessment data.

The NP faces many challenges in its application related to time constraints, understaffing, lack of training, and challenges in integrating and analyzing large volumes of information [3, 24, 36]. While most nurses have been trained to apply NP in their care, studies have shown that its implementation in digital health systems is often not captured in daily practice [24], with flow-on effects for the continuity, quality, and safety of care [36–38]. These challenges arise when nurses’ clinical reasoning is absent from documentation, even if their actions are performed but not recorded.

The implementation of nursing electronic health records (N-EHRs) was initially expected to support NP through the creation of smarter and safer workflows to enhance care quality and alleviate administrative burden [39]. However, most commercially available N-EHRs do not provide support for NP [24, 39], and research on systems specifically designed to assist nurses in clinical decision-making (particularly in formulating NDs and care plans) remains limited [3, 24, 29, 40]. A recent study by Hants and colleagues on the integration of NP into digital health systems found that N-EHRs have not yet reached the level of sophistication required to fully capture the complexity of nursing care. Instead, they primarily focus on data storage and care planning documentation than operating as CDSSs that actively support NP. This limitation may pose a risk to patient safety [24].

CDSSs supporting the implementation of NP or “NP-CDSSs” are expected to enhance nursing decision-making, enabling patient care plans to be reasonably developed and allowing for the accurate assessment of NDs, thereby leading to effective nursing interventions being implemented and patient-care goals being reached [29, 39]. Given the importance of NP-CDSSs in supporting nursing care quality and documentation, as well as their limited implementation in care settings, providing guidelines that define the essential features of NP-CDSSs and enhance their design and implementation became a priority for nursing informaticians and software developers [23, 41]. The internationally accepted standard for NP-CDSSs (ICS4NP-CDSSs) was developed in 2016 by Müller-Staub and colleagues [29] as the “gold standard” for an accurate NP documentation framework and guidance for the development of NP-CDSSs to be included within N-EHRs. The recommended features in this standard encompass various aspects, including ergonomics — that is, interfaces that are intuitively organized, visually accessible, and support efficient and error-free use in real-world clinical settings, the integration of NP-CDSSs within other N-EHR modules, and essential functionalities deemed mandatory to support NP and improve NP-CDSS implementation.

Several reviews have examined CDSSs in relation to NP. Three reviews [3, 23, 42], published in 2017, 2013, and 2017, respectively, included studies published up to 2013. However, aside from being outdated, they primarily focused on single-condition CDSSs, with little to no emphasis on NP-CDSSs designed for patients with multiple conditions, nor did they assess NP-CDSS features in relation to the ICS4NP-CDSS standard. More recently, two reviews [24, 40] were published in 2021 and 2023, respectively. Review [40] examined the impact of automated information processing on decision-making but did not analyze NP-CDSS features. In the same way, review [24] explored the integration of NP into digital health systems, focusing on electronic medical records, electronic care plans, and CDSSs, but without specifically addressing NP-CDSSs or their alignment with the ICS4NP-CDSS standard.

To our knowledge, since the introduction of the ICS4NP-CDSS standard in 2016, no review has systematically assessed whether NP-CDSSs have been developed in compliance with this framework. These gaps highlight the need for a timely scoping review to update and map existing knowledge on NP-CDSSs while evaluating their adherence to the ICS4NP-CDSS standard.

The objective of our scoping review is thus twofold: first, to update existing knowledge on the current state of NP-CDSS implementation and evaluation, including an assessment of nurses’ perceived barriers and facilitators to implementation; and second, to determine the extent to which current NP-CDSSs align with the ICS4NP-CDSS standard.

## Methods

### Recommendations of the internationally consented standard for NP-CDSSs

One objective of this review is to compare the features of published NP-CDSSs with the recommendations of the internationally consented standard for NP-CDSSs (ICS4NP-CDSSs). Developed through a multistep process, this standard comprises 25 criteria established by expert consensus. To enhance clarity, we proposed organizing these criteria into eight categories (see Table 2), providing a structured, yet comprehensive, presentation of all 25 criteria.

**Table 2:** Key criteria of the internationally accepted standard for NP-CDSSs (2016)

1.	NP-CDSSs place the NP at the core of nursing information and documentation, ensuring the use of SNLs.
2.	NP-CDSSs support nurses by integrating all phases of NP, offering accurate and evidence-based nursing diagnoses, interventions, and outcomes, while also allowing free text entry. Additionally, NP-CDSSs provide pre-structured, standardized, logical, and knowledge-based linkages between all NP steps.
3.	Nursing diagnoses, interventions, and outcomes within NP-CDSSs are linked to measurement instrument results (*e.g.,* pain, delirium, and pressure ulcer scores), as well as medical, allied healthcare, interdisciplinary diagnoses, interventions, outcome data, and biomedical scores.
4.	NP-CDSSs support comprehensive nursing documentation, covering physical, psychosocial, functional, and environmental aspects, while enabling the modification of care plans and clinical pathways. This flexibility ensures timely updates based on emerging evidence, such as changes in clinical guidelines or protocols.
5.	NP-CDSSs establish connections between NP elements with staffing levels and workload.
6.	NP-CDSSs generate alerts for missing abnormal biomedical results in NP documentation, or for conflicting data entries/information.
7.	NP-CDSSs provide efficient and user-friendly NP documentation, promoting usability and seamless integration into nurses’ workflows.
8.	NP-CDSSs contain coded and standardized concepts to facilitate data collection, sharing, and research.

### Design of the scoping review

We conducted a scoping review, as this approach is particularly well-suited for mapping key concepts within a research area, identifying the main types of available evidence, and uncovering gaps in the existing literature. Given these objectives, following the recommendations of Arksey and O’Malley [43], later refined by Levac and colleagues [44], this review was conducted in five stages: 1) identifying the research questions, 2) identifying relevant studies, 3) selecting relevant studies, 4) charting the data, and 5) collating, summarizing, and reporting the results. To ensure rigorous and transparent reporting, we followed the Preferred Reporting Items for Systematic reviews and Meta-Analyses extension for Scoping Reviews (PRISMA-ScR) checklist [45].

### Identifying the research questions

The guiding questions were established according to the PICO strategy (P = Problem, I = Intervention, C = Comparison, O = Outcomes) [46] with NP corresponding to the P, the use of an NP-CDSS corresponding to the I, the “ICS4NP-CDSSs” proposed by Müller-Staub and colleagues [29] corresponding to the C, and the identification of NP-CDSS characteristics in addition to nurses’ reported qualitative findings relative to their experience with an NP-CDSS corresponding to the O.

The research questions (RQ) are as follows:

#### RQ1:

What are the characteristics of NP-CDSSs published in the last decade, including the conception year, the country, the clinical settings covered, whether the system was commercially available or not, the system maturity level, the underlying decision support methodology implemented (knowledge-based (KB) or non-knowledge-based), whether data was collected only by nursing input or by automatic retrieval, the presence of a prioritization method to choose the most relevant NDs, and the outcomes measured if available?

#### RQ2:

Which functional features of ICS4NP-CDSS have been implemented in the retrieved NP-CDSSs?

#### RQ3:

What barriers and facilitators to NP-CDSS implementation have been reported by nurses?

### Identifying relevant studies

#### Eligibility criteria

Studies eligible for inclusion were primary original studies on CDSSs supporting NP and used in hospital settings. As three previous reviews [3, 23, 42] included studies published up to 2013, we conducted our review by including studies published in peer-reviewed journals from January 2013 to February 2024, with no language restrictions. Studies that involved non-electronic decision support, studies in which CDSSs were used by health professionals other than nurses, studies not conducted in hospital settings, or studies in which CDSSs did not significantly support NP steps (*e.g.*, covering no or only one domain) were excluded. Reviews and protocols were also excluded.

#### Search strategy

We chose to only search PUBMED CENTRAL and MEDLINE due to its extensive coverage of high-quality, peer-reviewed research in biomedical and health sciences (covering over 93.4% of publications) [47, 48]. Keywords and search terms were developed in accordance with the Peer Review of Electronic Search Strategies (*PRESS*) Guidelines Evidence-Based Checklist [49] by two researchers with expertise in the field of nursing informatics (CAK and BS). The query framework and its delineated concepts were defined, and related terms were harvested to construct a comprehensive list of synonyms. Two authors (CAK and JB) developed it by identifying and extracting controlled vocabulary terms (*i.e*., MeSH terms) and in-text synonyms (*i.e*., “common terms”) that best captured the research question and key search components. The strategy also involved avoiding syntax errors, comparing the query against existing literature, and fine-tuning it to maximize relevant results. The full query is provided in Supplementary Material 1.

### Selecting relevant studies

All titles and abstracts were screened by the first author (CAK) and by two of the co-authors (AS and JR), who performed an independent second review each on half of the identified references. The three authors (CAK, AS, and JR) rated each article as “relevant”, “potentially relevant” or “not relevant” according to both inclusion and exclusion criteria, and for each paper retrieved, we obtained a pair of ratings. Disagreements were resolved by consensus after discussion and reading of the full text. During the reading of the selected articles in full length, citations of works, not considered in the initial search, were also collected.

### Charting the data

Three authors (CAK, AS, and JR) collectively developed a data-charting form in which the variables to be extracted were purposefully chosen to answer the research questions. First, they independently extracted data from the same ten studies, randomly chosen among the retrieved studies, using the data-charting form to check whether the form was adapted and whether the data extraction method could be consistently applied. The data-charting form was then amended, and the data extraction method was refined (inter-rater agreement of 0.80, as measured by Cohen’s kappa coefficient [50]) and applied by the same three authors to all retrieved articles.

The following data were extracted for each article: study title, authors, publication year, purpose, study analysis, data collection method, NP-CDSS name and description, conception year, country, clinical settings covered, whether the system was commercially available or not, decision-support method implemented (knowledge-based or non-knowledge-based NP-CDSSs), data collected only by nursing input or by automatic retrieval, NP-CDSS maturity level based on 6 levels (preconception, conception, laboratory pre-implementation, pilot evaluation, post-implementation validation and usability studies when available) defined in Table 3, measured outcomes when available, prioritization method to choose the most relevant NDs when available, CDSS features as recommended by the ICS4NP-CDSSs, and qualitative findings relative to nurses’ experience with NP-CDSSs (barriers and facilitators for NP-CDSS implementation) when mentioned.

**Table 3:** Definitions of NP-CDSSs maturity levels

Preconception	Understanding the clinical context, user requirements, and problems through discussions with stakeholders and user group representatives.
Conception	Designing the CDSS by defining its components, data sources, algorithms, and user interfaces, followed by coding and integrating these elements.
Laboratory pre-implementation	Verifying if the CDSS functions correctly, with proper linkage to the EHR and assessing whether the alerts generated by the CDSS are clinically relevant.
Pilot evaluation	Identifying issues, refining the design, and gathering data on its effectiveness before larger-scale deployment.
Implementation	Using NP-CDSS in real clinical workflow by the end users, after installation
Post-implementation validation	Monitoring performance, adjusting clinical rules, and ensuring alert accuracy through ongoing update after implementation.
Usability studies	Assessing the CDSS’s ease of use, ensuring it integrates smoothly into clinical workflow to improve patient care and decision-making.

### Collating, summarizing and reporting the results

The findings of this review were displayed in four different tables. Findings relative to the study title, authors, publication year, purpose, study analysis, data collection methods, and NP-CDSS name and description are collected and displayed in one table. The findings related to the general characteristics of the retrieved NP-CDSSs related to the conception year, country, clinical settings covered, whether the system was commercially available or not, decision support method, data collected only by nursing input or by automatic retrieval, NP-CDSS maturity level, measured outcomes relative to nurses or to patients (if mentioned), and prioritization system are displayed in another table. Features related to ICS4NP-CDSSs are presented in a third table. Finally, qualitative findings related to barriers and facilitators of NP-CDSS implementation were synthesized, and presented according to the evaluation framework Human, Organization and Technology-fit factors (HOT-Fit) for health information systems [51] and are presented in a last table. 

Authors labeled features “Yes” when the feature was clearly present in the studied system, “No” when it was not present, “Not Reported” when no element relative to this feature was reported in the studies and “Not clear” when the mentioned elements were not clear enough to assess the exact value of the feature.

### Data analysis

The data were analyzed by four of the authors (CAK, AS, JR, and BS) using descriptive statistics.

### Ethical considerations

For secondary data analysis, which was conducted on non-personal data, ethics approval was not required for this scoping review.

## Results

### General description of the included studies

**Fig. 1:**
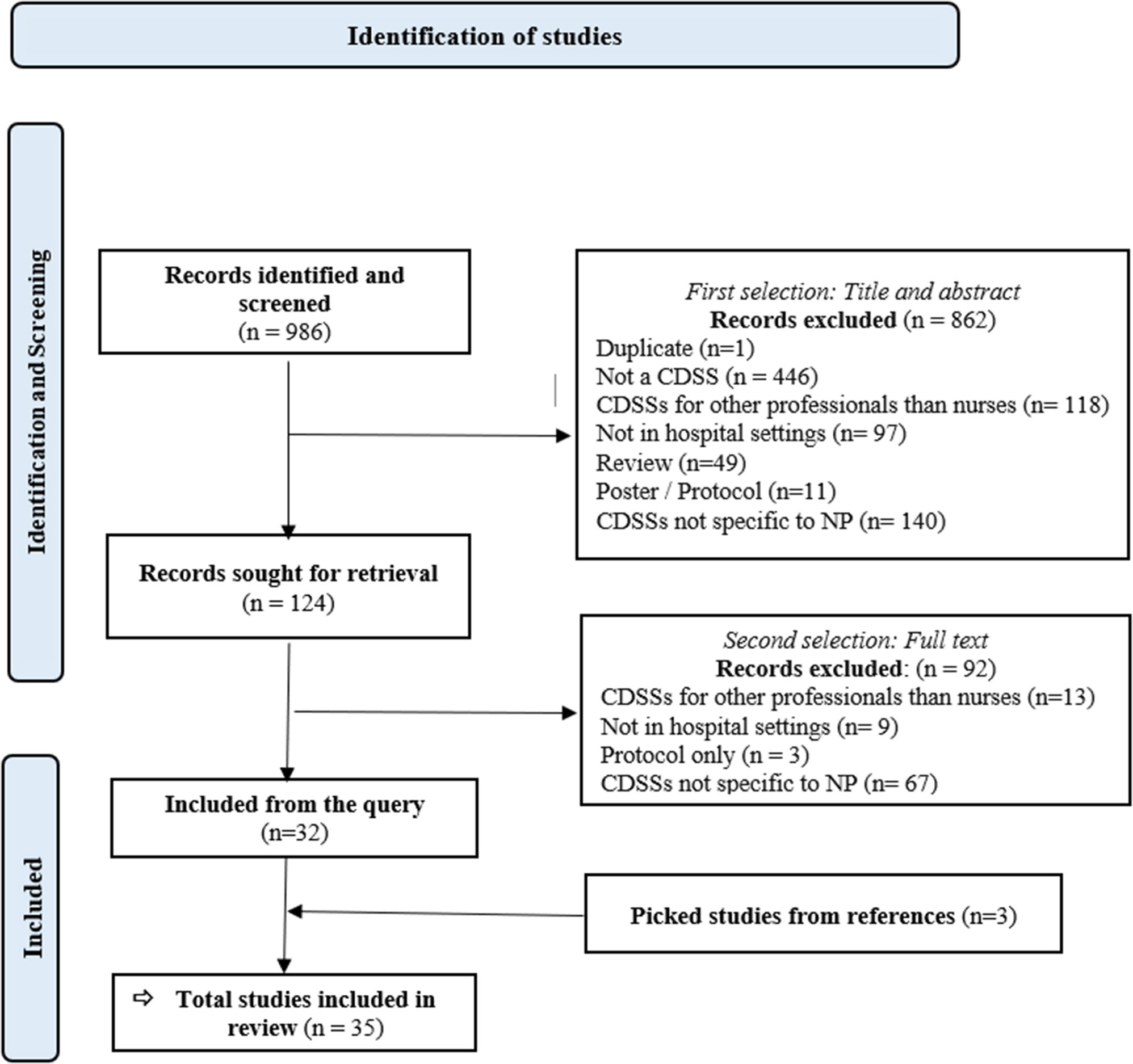
Prisma flow chart to represent the selection of relevant studies

Of the 986 articles retrieved by the query, 124 were selected after applying the inclusion and exclusion criteria. Among these, 92 articles were subsequently excluded after reading the full text, and three articles resulting from the “reverse search” of the references of included articles were added, leading to a final selection of 35 studies related to 21 different NP-CDSSs as displayed in Fig. 1.

Twenty-two of the studies identified (63%) were descriptive [25, 36, 52–71], seven (20%) [26, 39, 72–76] were designed as pre-post evaluations, and six (17%) [77–82] were randomized controlled trials related to two systems (HANDS [77–80, 82] and Hosseini et al [81]). (The details of the studies are summarized in Table 4).

**Table 4:** Studies Characteristics- year of publication, purpose, design, data collection methods and relative CDSSs

A**uthor(s)**	Title	Publication year	Study purpose	Study analysis	Data collection methods	Name of CDSSs and description
Szydłowska-Pawlak et al.	Nursing Care Plan for a Newborn with the Defect of Congenital Gastroschisis in the Postoperative Period Using ICNP(TM) and the Dedicated Software	2022	Formulate a care plan for a newborn with diagnosed congenital defect gastroschisis in the postoperative period, using the International Classification for Nursing Practice within the nursing documentation and decision support system, the “ADPIECare Dorothea” software.	Descriptive	Qualitative methods	“ADPIECare Dorothea” software applies to neonatal care and covers all the phases of the nursing process except implementation and evaluation.
Zhai et al.	Transition to a new nursing information system embedded with clinical decision support: a mixed-method study using the HOT-fit framework	2022	Investigate nurses’ perceptions and experiences with transition to a new nursing information system (Care Direct) 2 years after its first introduction.	Descriptive	Mixed methods	Care Direct applies to clinical units and covers all the phases of the nursing process except evaluation.
Sônia Regina Wagner de Almeida et al.	Computerized nursing process in the Intensive Care Unit: ergonomics and usability	2016	Analyze the ergonomics and usability criteria of the Computerized Nursing Process based on the International Classification for Nursing Practice in the Intensive Care Unit according to International Organization for Standardization.	Pretest-posttest	Quantitative methods	CNP applies to critical care and covers all the phases of the nursing process except implementation and evaluation.
Dal Sasso GM, Barra DC.	Cognitive Workload of Computerized Nursing Process in Intensive Care Units	2015	Measure the cognitive workload to complete printed nursing process versus computerized nursing process from International Classification Practice of Nursing in intensive care units.	Pretest-posttest	Quantitative methods	
Daniela Couto Carvalho Barra	Warning systems in a computerizednursing process for Intensive Care Units	2013	Establish associations between the data and information that are part of a Computerized Nursing Process according to the ICNP® Version 1.0, indicators of patient safety and quality of care.	Descriptive	Qualitative methods	
Dal Sasso GT et al.	[Computerized nursing process: methodology to establish associations between clinical assessment, diagnosis, interventions, and outcomes]	2013	Development of a Computerized Nursing Process for the Intensive Care Unit.	descriptive	Quantitative methods	
Paese et al.	Structuring methodology of the Computerized Nursing Process in Emergency Care Units	2018	Structure the Computerized Nursing Process using the International Classification for Nursing Practice (ICNP®) version 2.0 to emergency care units in a computerized structure.	Descriptive	Qualitative methods	e-RUE® project applies to emergency care and covers all the phases of the nursing process except implementation and evaluation.
Bertocchi L et al.	Nursing Diagnosis Accuracy in Nursing Education: Clinical Decision Support System Compared With Paper-Based Documentation-A Before and After Study	2023	Compare the nursing diagnostic accuracy, satisfaction, and usability of a computerized system versus a traditional paper-based approach.	Pretest-posttest	Quantitative methods	Florence applies to clinical units.
Lopez KD et al.	Conducting a representative national randomized control trial of tailored clinical decision support for nurses remotely: Methods and implications	2022	Compare 4 randomly assigned CDS format groups (text, text table, text graphs, tailored to subject’s graph literacy score) for effects on decision time and simulated patient outcomes.	Randomized trial	Quantitative methods	HANDS applies to clinical units and covers phases covers all the phases of the nursing process with elements not clear about assessment prompting Nursing Diagnoses
Cho H et al.	Assessing the Usability of a Clinical Decision Support System: Heuristic Evaluation	2022	an example application of a systematic evaluation method that uses clinician experts with human-computer interaction (HCI) expertise to evaluate the usability of an electronic clinical decision support (CDS) intervention prior to its deployment in a randomized controlled trial.	Randomized trial	Mixed methods	
Stifter J et al.	Acceptability of Clinical Decision Support Interface Prototypes for a Nursing Electronic Health Record to Facilitate Supportive Care Outcomes	2018	Determine the acceptability, usefulness, and ease of use for four nursing clinical decision support interface prototypes.	Randomized trial	Quantitative methods	HANDS applies to clinical units and covers phases covers all the phases of the nursing process with elements not clear about assessment prompting Nursing Diagnoses.
Karen Dunn Lopez, PhD et al.	Toward a More Robust and Efficient Usability Testing Method of Clinical Decision Support for Nurses Derived From Nursing Electronic Health Record Data	2017	Develop methods for rapid and simultaneous design, testing, and management of multiple clinical decision support features to aid nurse decision-making.	Descriptive	Qualitative methods	
Keenan GM et al.	Toward Meaningful Care Plan Clinical Decision Support: Feasibility and Effects of a Simulated Pilot Study	2017	Compare three experimental CDS format groups (text, text + graph, and text + table) to control (No CDS) on RNs’ adoption of best practices and care-planning time and to examine the effects of numeracy and graph literacy on the adoption rates and time.	Randomized trial	Quantitative methods	
Vanessa E. C. Sousa, et al.	Use of Simulation to Study Nurses’ Acceptance and Nonacceptance of Clinical Decision Support Suggestions	2015	Explore reasons for nursing acceptance and non-acceptance of a prototype care plan suggestions during a high fidelity clinical simulation experience.	Randomized trial	Quantitative methods	
Febretti et al.	Evaluating a Clinical Decision Support Interface for End-of-Life Nurse Care	2014	Designing and testing a CDSS interface embedded within a nurse care planning and documentation tool. (four prototypes based on different CDSS feature designs in simulated end-of-life patient handoff sessions with a group).	Pretest-posttest	Quantitative methods	
Silva Jr MG et al.	Software for systematization of nursing care in medical units	2018	Describe the development of a software prototype to apply the nursing process in clinical units of a general hospital, and assess its usefulness.	Descriptive	Qualitative methods	INFOSAE applies to clinical units and covers all the phases of the nursing process.
Zega et al.	Development and validation of a computerized assessment form to support nursing diagnosis	2013	Describe the development and validation of the Nursing Assessment Form, within a clinical nursing information system, to support nurses in the identification of nursing diagnoses.The aim of this study was to develop a computerized nursing assessment form to facilitate the diagnostic reasoning of nurses to identify nursing diagnoses and then estimate its content validity.	Descriptive	Qualitative methods	NAF applies to clinical units and covers only the assessment and Problem Identification/Diagnosis phases of the nursing process.
José Janailton de Lima et al.	Computerized nursing process: development of a mobile technology for use with neonates	2018	Build a mobile technology to assist nurses during data collection, diagnostic reasoning, and identification of interventions in neonates.	Descriptive	Qualitative methods	NATUS applies to neonatal care and the available elements point to covering the interventions phase
Te-Hui Hao et al.	Nursing process decision support system for urology ward	2013	Develop a nursing process decision support system based on three clinical pathways, including benign prostatic hypertrophy, inguinal hernia, and urinary tract stone.	Descriptive	Mixed methods	NPDSS applies to urology and Diagnosis and evaluation phases of the nursing process.
Zhai et al.	How Nurses Develop Standardized Care Plans Under a Clinical Decision Support System: A Mixed-Methods Study	2023	Investigate acute care nurses’ practices and experiences of care planning within a clinical decision support system embedded with standardized nursing languages.	Descriptive	Mixed methods	Nu-CDSS applies to clinical units and covers all the phases of the nursing process except evaluation.
Zhai Y ey al.	Barriers and facilitators to implementing a nursing clinical decision support system in a tertiary hospital setting: A qualitative study using the FITT framework	2022	Explore barriers and facilitators to the implementation of a CDSS from the perspective of nurses.	descriptive	Qualitative methods	Nu-CDSS applies to clinical units and covers all phases the nursing process.
Yongxin ZHAOa et al.	Development and Implementation of Pediatric Nursing-Clinical Decision Support System for Hyperthermia: A Pre- and Post-Test	2021	This study consisted of two stages. The first stage was PaedN-CDSS- hyperthermia development, which was then continued by its preliminary examination of the implementation before (1–31 August 2018) and after (1–31 December 2019) using PaedN-CDSS-Hyperthermia.	Pretest-posttest	Quantitative methods	PedN-CDSS-Hyperthermia applies to pediatrics unit and covers all the phases of the nursing process.
Dos Santos Diogo RC et al.	Diagnostic concordance among nursing clinical decision support system users: a pilot study	2023	Analyze the nursing diagnostic concordance among users of a clinical decision support system, The Electronic Documentation System of the Nursing Process of the University of São Paulo (PROCEnf-USP®), structured according to the Nanda International, Nursing Intervention Classification and Nursing Outcome Classification (NNN) Taxonomy.	descriptive	Qualitative methods	PROCEnf-USP applies to clinical units and covers all the phases of the nursing process except evaluation.
Regina Célia dos Santos Diogo, et al,	Evaluation of the Accuracy of Nursing Diagnoses Determined by Users of a Clinical Decision Support System	2021	Analyze the accuracy of nursing diagnoses determined by users of a clinical decision support system and to identify the predictive factors of high/moderate diagnostic accuracy.	Descriptive	Quantitative methods	
Oliveira NB et al.	Quality of the documentation of the Nursing process in clinical decision support systems	2021	Compare the quality of the Nursing process documentation between the version 1 and the version 2 of versions of PROCEnf-USP.	Pretest-posttest	Quantitative methods	
Heloisa Helena Ciqueto Peresa et al.	Usability Testing of PROCEnf-USP: A Clinical Decision Support System	2015	Evaluate the interactions between registered nurses and the Decision Support Systems, and to explore how they impact RN decision-making.	Descriptive	Quantitative methods	
Kuei-Fang Ho et al.	Design and evaluation of a knowledge-based clinical decision support system for the psychiatric nursing process	2021	Design and evaluate a knowledge-based clinical decision support system for the psychiatric nursing process.	Pretest-posttest	Quantitative methods	Psy-KBCDSS applies to psychiatry and covers the assessment and the problem Identification/Diagnosis phases of the nursing process.
Kenya de Lima Silva et al.	Software development to support decision making in the selection of nursing diagnoses and interventions for children and adolescents	2015	Report the development of software to support decision-making for the selection of nursing diagnoses and interventions for children and adolescents, based on the nomenclature of nursing diagnoses, outcomes and interventions of a university hospital in Paraiba.	Descriptive	Qualitative methods	SISPED applies to pediatrics and covers all the phases of the nursing process except evaluation.
Lee S et al.	Implementation of Structured Documentation and Standard Nursing Statements: Perceptions of Nurses in Acute Care Settings	2019	Describe perceptions of nurses using SYSTEM featuring standard nursing statements and structured documentation.	Descriptive	Quantitative methods	SYSTEM applies to clinical units and covers the assessment, problem identification/Diagnosis and implementation phases of the nursing process.
da Costa C, Linch GFDC.	Implementation of Electronic Records Related to Nursing Diagnoses	2018	Implement electronic health records regarding the NP in the computerized health management system in a hospital.	Descriptive	Qualitative methods	TASY applies to clinical units and covers all the phases of the nursing process except evaluation.
Bente CHRISTENSEN et al.	Clinical Decision Support: Evaluating the Development of a Tool for Nurses	2023	Evaluate and document the development and use of VAR Healthcare and ensure that the tool was evolving in the right and meaningful direction for an advanced clinicaldecision support tool for nurses.	Descriptive	Qualitative methods	VAR applies to clinical units and covers only Plan/Interventions phases of the nursing process.
Alsadat Hosseini F et al.	Using Newly Developed Software to Enhance the Efficiency of the Nursing Process in Patient Care: A Randomized Clinical Trial	2021	Determine the effectiveness of applying newly developed nursing process software on the efficiency of the nursing process in patient care.	Randomized trial	Quantitative methods	(Unknown name) applies to clinical units and covers all the phases of the nursing process.
Kobra Parvan et al.	Attitude of nursing students following the implementation of comprehensive computer-based nursing process in medical surgical internship: a quasi-experimental study	2021	Evaluate nursing students’ attitudes towards the nursing process software.	Descriptive	Quantitative methods	
Arzu Akman Yılmaz et al.	Development and Implementation of the Clinical Decision Support System for Patients With Cancer and Nurses’ Experiences Regarding the System	2017	Develop and implement the CDSS software for nurses working with patients with cancer and to explore the nurses’ experiences regarding the system.	Descriptive	Mixed methods	(Unknown name) applies to oncology and covers all the phases of the nursing process except implementation and evaluation.
Pei-Hung Liao et al.	Applying artificial intelligence technology to support decision-making in nursing: A case study in Taiwan	2015	Application of artificial intelligence to help nurses address problems and receive instructions through information technology.	Descriptive	Quantitative methods	(Unknown name) applies to clinical units and covers Assessment and problem identification/diagnosis phases of the nursing process.

Phase 1: Assessment; Phase 2: Problem Identification/Diagnosis; Phase 3: Care Plan/Interventions; Phase 4: Implementation; Phase 5: Evaluation

### General characteristics of the identified NP-CDSSs

Among the 21 identified systems, three systems (14%) (HANDS, CNP, and PROCEnf-USP) [57, 72, 74, 77–80, 82] were described in the late 1990s and represented almost half of the included studies (15 out of 35 studies). For the 18 remaining systems (86%), the conception year spanned between 2005 and 2023, with a relatively constant creation rate since 2012 (approximately two new systems per year). Most of the 21 systems originated from Brazil (7 systems) (33%) [39, 54, 56, 58, 60, 63, 68, 72], followed by Taiwan (3 systems) (14%) [25, 71, 76], China (3 systems) (14%) [53, 61, 62, 75], Italy (2 systems) (9%) [26, 59] and 1 system (5%) from each of the USA [57, 74, 77, 79, 80, 82], Turkey [36], Poland [52], Korea [67], Norway [69] and Iran [70, 81].

For the clinical settings, 12 systems (57%) [26, 39, 53, 57–59, 61–65, 67–71, 74, 77–83] were intended for clinical wards (Medical/Surgical), one (5%) [54, 55, 72, 73] for critical care, and the remaining systems (38%) were intended for specialized services, *e.g*., Emergency, Pediatrics, Neonatology, Urology, Oncology, and Psychiatry [25, 36, 52, 56, 60, 66, 75, 76].

Five systems (24%) provided enough details to determine that they were commercialized (CareDirect [53], Nu-CDSS [61], VAR [69], ADPIECare Dorothea [52], Florence [26])

Almost all systems were knowledge-based CDSSs with only one system (5%) [71] using machine learning algorithms to predict common NDs [71]. Four systems (19%) [36, 56, 71, 75] offered some automatic data retrieval, whereas nurses were required to manually complete each criterion of the assessment sheets in the remaining systems. Additionally, none of the systems supported nurses in prioritizing NDs.

None of the 21 systems reported having undertaken a preconception study, seven (33%) described only a conception study without further validation [25, 36, 52, 56, 58, 60, 66], seven (33%) reported a pre-implementation validation study (in laboratory settings or simulation) [57, 59, 63, 64, 68, 71, 73, 74, 76–78, 82], five (24%) reported a pilot evaluation study [39, 53, 64, 65, 68, 75, 76, 81], and four (19%) underwent a post-implementation analysis [26, 61, 67, 69]. Eleven systems (52%) [25, 36, 39, 53, 54, 57, 61, 64, 65, 69, 72, 74, 75, 77–79] reported at least one usability study, among which eight provided qualitative findings regarding system usability [53, 62, 64, 65, 67, 74, 77, 78] which allowed to identify the barriers and facilitators to implementation described in in this study. Four systems (19%) reported outcome measurements [39, 64, 73, 81]. These outcome measurements were quantitative and mainly focused on various aspects, including documentation quality [39], NDs accuracy [64], cognitive burden [73], and NP quality [81]. No study reported measures related to patient outcomes. All results are detailed in Table 5.

**Table 5:**
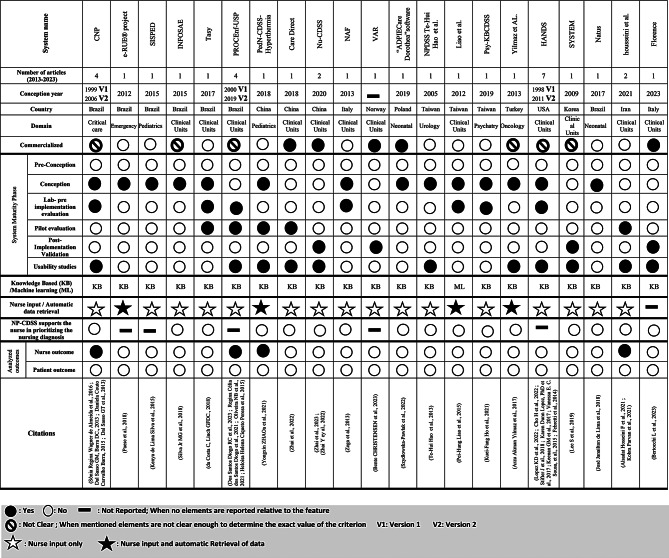
General characteristics of NP-CDSSs in the included studies

### Compliance of NP-CDSSs with ICS4NP-CDSSs

The following section details the characteristics derived from the ICS4NP-CDSSs standard.

### Coverage of NP phases

Among the 21 systems, 19 (90%) [25, 26, 36, 39, 52, 53, 55, 58, 59, 62–64, 67, 68, 71, 72, 76, 81, 83, 84] covered the assessment phase, 21 (100%) [25, 26, 36, 39, 52, 53, 55, 57–59, 62–64, 67–69, 71, 72, 76, 78, 80, 81, 83, 84] covered the problem/diagnosis phase, 16 (76%) [26, 36, 52, 53, 55, 56, 58, 60–62, 64, 65, 68–70, 72, 74, 75, 77–82] covered the care plan/interventions phase, 12 (57%) [26, 53, 56, 58, 60–62, 64, 65, 68, 70, 74, 75, 77–82] covered the implementation phase and only 8 (38%) [26, 39, 53, 62, 63, 65, 70, 74, 77–82] covered the evaluation phase.

### Sequential implementation phases

Of the 19 systems that covered the assessment phase, all 19 (100%) also covered the ND phase. Additionally, 14 systems (73%) [26, 36, 39, 52, 53, 55, 56, 58, 60–65, 68, 70, 72, 75, 77, 79, 81] covered the sequence [Assessment-ND-Planning], 10 systems (52%) [26, 36, 39, 53, 56, 58, 60–65, 70, 75, 77, 81] covered the sequence [Assessment-ND-Planning-Implementation], and only seven systems (36%) [26, 39, 53, 58, 61–65, 70, 75, 81] covered the complete nursing process sequence including evaluation.

### Automatic linkages between phases

In 17 out of 21 systems (81%) [25, 36, 39, 52, 53, 55, 56, 58, 59, 61–67, 70–73, 75, 76, 81], NP-CDSSs provided diagnostic support when characteristics, related factors, or risk factors were entered. Automatic linkages between NDs and intervention steps were present in 15 systems (71%) [39, 52–56, 58, 60–70, 72–75, 77–82], with 10 systems (48%) [39, 53, 57, 58, 61–68, 70, 74, 75, 81] reporting linkages between NDs/interventions and patient outcomes. Additionally, five systems (24%) [25, 58, 67, 75, 77–80, 82] included outcome criteria related to NDs at the evaluation step.

### Other features

All systems but one (95%) [75] used defined SNLs: nine (43%) used NANDA/NIC/NOC [25, 26, 36, 39, 57–60, 64, 65, 68, 70, 71, 74, 76–79, 81, 82], with NANDA providing nursing diagnoses [26], NIC defining nursing interventions, and NOC [31] specifying measurable patient outcomes. Three systems (14%) [36, 71, 76] used NANDA-I, six systems (28%) [52, 54, 56, 66, 67, 69, 72, 73] used ICNP [30], and one system (5%) [61, 62] used CCC [32, 33]. One system (5%) [53] used NMDS (Nursing Minimum Data Set) [85], which is a core set of essential nursing data elements used for research, policy-making, and healthcare management, ensuring standardized data collection across settings. All SNLs are integrated into SNOMED-CT [86].

ND used holistic assessment (*i.e.*, covering physical, psychosocial, functional and environmental aspects) in all but four systems (81%) [25, 26, 61, 62, 75].

The integration of NP-CDSSs with medical diagnoses, interventions or outcomes (e. g., automatically importing current ICD-10 diagnoses and recent surgical procedures of the patient into the nursing assessment) was found in five systems (24%) [36, 53–56, 72, 73, 75], whereas the integration of biomedical data (e. g., vital signs, lab results) from the hospital information system was clearly reported in two systems (10%) [36, 54, 55, 72, 73] out of the 21. The integration of the results of measurement instruments (such as pain assessment, pressure ulcer assessment, and fall risk assessment) within NP-CDSSs was reported in five systems (24%) [36, 53, 56, 58, 75]. The possibility for nurses to document a full text entry was offered by three systems (14%), mainly in the first (assessment) and last (evaluation) steps of the NP [39, 53, 59, 63–65].

One system (5%) [58] was able to support workload measurement or required staff qualifications, nine systems (43%) [53, 56, 57, 60–62, 66, 67, 69, 72, 74, 75, 77–80, 82] reported supporting the modification of interdisciplinary care plans when new evidence was available (it was not clear in the available literature whether new evidence was manually input or acquired from other information systems), and eight systems (38%) [54–58, 60, 66, 69, 72–75, 77, 78, 80, 82, 87–89] supported the ergonomic visualization of system processing (in particular, when the user could obtain and navigate through all needed information without scrolling between multiple screens or modules). Finally, none of the systems proposed automatic alerts about conflicting data, and only two (10%) offered alerts about missing data [53, 57, 74, 77–80, 82].

With reference to the best practice features for NP-CDSSs, the detailed results are shown in Table 6.

**Table 6:**
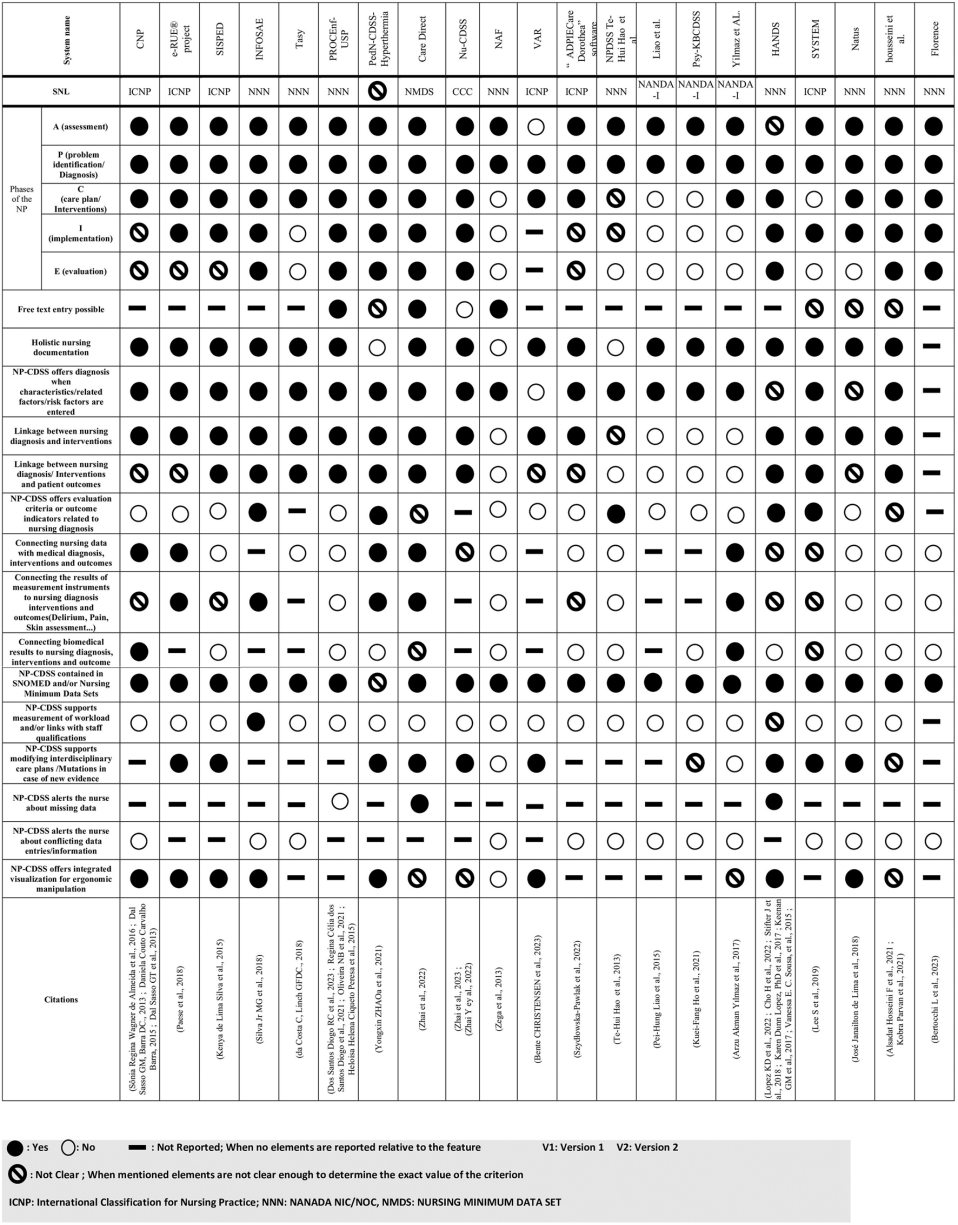
Features of NP-CDSSs relative to the International consented standard for NP-CDSSs

### Barriers and facilitators reported in usability studies

Barriers and facilitators reported by nurses regarding the implementation of NP-CDSSs were extracted from the qualitative studies included in the review [25, 36, 53, 57, 61, 62, 64, 67, 70, 72, 74, 75, 77, 79, 80, 84], and are presented in Table 7. The most frequently reported barriers included a lack of confidence in the systems [77, 78], a perceived lack of usefulness of NP-CDSSs in improving workflows [61, 67], the inability of NP-CDSSs to be flexible or able to capture complex and varied patient conditions [61, 67], a failure to prioritize recommended interventions leading to confusion [53, 61, 62], an increased documentation burden [61, 67], a lack of interoperability/integration [53, 62], issues related to system design and usability [53, 61, 62], poor integration of unit culture [61], insufficient training [53, 62], and a lack of technical support [53, 62]. The facilitators identified were primarily the expectation of improved care quality [77], more efficient nursing practice [67], well-designed user interfaces [67, 74], reminders [53, 62, 77], nurse involvement at all stages of the system development [64], comprehensive training [64, 65, 84], and management and technical support [53, 61, 62].

**Table 7:** Synthesis of usability barriers and facilitators according to the HOT-Fit framework

**Category as per HOT-Fit framework: Human**	**Barriers**	**Facilitators**
***Theme 1: *** *Perceived usefulness/intention to use/acceptance/confidence/satisfaction*	• Lack of perceived usefulness of applying individualized care as per the NP in a context of task-based routines acquired in the wards, traditions of narrative records and increasing load and time-constraints (Zhai et al., 2023) • Standardized interventions recommended by the system are perceived as generic and lacking context, hence with little value for individualized care practice (Zhai et al., 2023, Lee et al., 2019) • Sense of increasing burden with increased charting time and decreased bedside care time (Lee et al., 2019) • The most frequent reasons for refusing CDSS suggestions were disagreement and lack of confidence in the displayed information (Sousa et al., 2015)	• Nurses had more intention to adopt the system when they felt the suggestions were good for the patient (Sousa et al., 2015) • When the system automatically provided appropriate standard nursing statements in each phase, it helped nursing practice with efficiency, such as reducing charting time and avoiding missing necessary nursing care (Lee et al., 2019). • Nurses perceived that combining the assessment step and the evaluation step and combining the planning step and the implementation step could reduce overlapping and increase the efficiency of nursing practice (Lee et al., 2019) • Nurses should be involved at all stages of the NP-CDSS development (Diogo et al., 2021)
***Theme 2:*** *Critical* *evaluation/knowledge/training*	• Risk of nurses adopting the system’s suggestions without a critical evaluation, hence failing to identify problems and treatments not presented in the NP-CDSS (Keenan et al., 2017) • Preset standard nursing statements hindered creativity of nurses and freshness of nursing care, and made nurses’ perspective on patient care narrow and fixed. Nurses became passive as they just followed the automated system. (Zhai, Yu, Zhang, Qin, et al., 2022; Zhai, Yu, Zhang, & Zhang, 2022; Lee et al., 2019) • Insufficient training on system use (Zhai, Yu, Zhang, Qin, et al., 2022; Zhai, Yu, Zhang, & Zhang, 2022)	• Sufficient continuing education and experience with the system (Diogo et al., 2021; Ciqueto Peres et al., 2015)
**Category as per HOT-Fit framework: Organization**	**Barriers**	**Facilitators**
***Theme 1: *** *Communication/culture/management*	• Lack of a standard of practice at the organizational level led to inconsistent system use patterns among nurses, which affected the confidence and motivation of nurses to make Standardized Care Plans. (Zhai et al., 2023)	• To tackle the uncertainty during system use, nurses sought to form a unified use pattern within the ward as much as possible by communicating with each other or under the guidance of the head nurse. (Zhai et al., 2023)
***Theme 2: *** *Follow up service, technical support, continuous improvement*	• Lack of collaboration/cooperation from IT staff (Zhai, Yu, Zhang, Qin, et al., 2022; Zhai, Yu, Zhang, & Zhang, 2022)	• The system continuously improves in response to user needs/feedback (Zhai, Yu, Zhang, Qin, et al., 2022; Zhai, Yu, Zhang, & Zhang, 2022)
**Category as per HOT-Fit framework: Technology**	**Barriers**	**Facilitators**
***Theme 1: *** *Ease of use/ergonomics/visualization/ functions*	• Complexity of assessment templates/many items to be filled not adapted to all patients (Zhai, Yu, Zhang, Qin, et al., 2022; Zhai, Yu, Zhang, & Zhang, 2022) • Shift handover summary template containing too many elements that are not hierarchized by importance (Zhai, Yu, Zhang, Qin, et al., 2022; Zhai, Yu, Zhang, & Zhang, 2022) • Because of the large number of interventions in the built-in knowledge base of the NP-CDSS, nurses were confused about how to choose between them, and there was a certain randomness among nurses when developing care plans (Zhai et al., 2023) • Recording care plans not implemented increases cognitive burden and workload of nurses. According to participants, plans not implemented are verbally transmitted to nurses in the following shift (Lee et al., 2019). • Some nursing tasks could not be properly represented with the standardized interventions recommended by the NP-CDSS, leading to perplexities among nurses. There was no place for nurses to type in additional interventions by free text in the care planning interface, thus compromising the accuracy of the care plan (Zhai et al., 2023).	• A reason for NP-CDSS recommendation acceptance was linked to using visuals implying a sense of urgency (e. g., red blinking button) (Sousa et al., 2015) • Interventions automatically recommended by the system can serve as a reminder (Zhai, Yu, Zhang, Qin, et al., 2022; Zhai, Yu, Zhang, & Zhang, 2022) • Well-designed NP-CDSS interfaces available to nurses at the point of care that are displayed during the decision-making workflow can influence care plan changes that may yield better patient outcomes. (Febretti et al., 2014) • Presenting multiple NP-CDSS features in a single window appears more effective than letting users “cherry pick” information they want to visualize. (Febretti et al., 2014) • Nurses have to be able to freely enter text in any phase if needed (Lee et al., 2019)
***Theme 2*** *: Integration with other systems*	• NP-CDSS not linked with laboratory or medical data, and does not update its information and recommendation accordingly (Zhai, Yu, Zhang, Qin, et al., 2022; Zhai, Yu, Zhang, & Zhang, 2022)	

## Discussion

Several reviews have been published on NP-CDSSs [3, 23, 24, 40, 42] but they have notable limitations. The older ones [3, 23, 42] only analyzed studies published up to 2013 and primarily focused on single-condition CDSSs, with little to no consideration given to NP-CDSSs addressing patients with multiple conditions. Additionally, none of these reviews, whether older or most recent [24, 40], evaluated NP-CDSS features in relation to the ICS4NP-CDSS standard. To bridge these gaps, we conducted a scoping review to *update* and *complete* the existing knowledge on NP-CDSS implementation and evaluation. This scoping review aims to map the features of NP-CDSS, identify the available evidence, and highlight gaps in the existing literature about NP-CDSSs. To our knowledge, this is the first scoping review to focus on NP-CDSSs published since 2013 and the first to compare the features of the retrieved systems with the ICS4NP-CDSS standard published in 2016 [29].

A combined analysis of our findings revealed three areas requiring significant improvement in NP-CDSS design and implementation: 1) Enhancing trust and perceived usefulness of NP-CDSSs, while preserving nurses’ critical thinking, 2) Reducing documentation burden, 3) Ensuring compliance with ICS4NP-CDSSs. Addressing these challenges is essential to advancing the development and implementation of NP-CDSSs in clinical practice.

### Enhancing trust, perceived usefulness, and preserving nurses’ critical thinking

The qualitative analysis of barriers in the original articles raises concerns about the level of trust and the perceived usefulness of NP-CDSS among nurses. Although research on NP-CDSSs lags behind studies on CDSSs designed for physicians, particularly in supporting diagnosis and recommendations, NP-CDSS implementation faces similar challenges regarding trust and confidence in the information provided, factors that may hinder adoption [61, 77, 90]. Qualitative evidence from nurses highlights key facilitators for successful NP-CDSS implementation, emphasizing the need to (i) involve nurses throughout all phases of an NP-CDSS project to ensure their needs and expectations are addressed [53, 91], and (ii) ensure that NP-CDSS recommendations are both valid and appropriate for patients, thereby improving quality, efficiency and preventing missed care [67, 77, 78]. Achieving this requires rigorous system evaluation before implementation [3], and advancing study designs through more sophisticated methodologies, expanded multisite research, and increased funding for NP-CDSS research [3]. Notably, the available details in the retrieved articles did not reveal any significant differences between commercial and non-commercialized NP-CDSSs.

Moreover, trust in NP-CDSSs could be strengthened if studies demonstrated improvements in patient and nursing outcomes. Unfortunately, none of the systems examined within our review period assessed patient outcomes related to NP-CDSS use, and only a few [39, 64, 73, 81] reported nursing outcomes, which aligns with findings from other reviews [3, 23].

A key driver for promoting the implementation of NP-CDSS is the integration of the NP framework into regulatory policies for N-EHR design. Regulatory frameworks can play a pivotal role in fostering the development and clinical implementation of NP-CDSSs [92], particularly in regions where their use is mandated. A notable example is Minnesota’s recommendation to incorporate the Omaha System classification into nursing electronic health record (N-EHR) platforms [93]. In our review, Brazil, Taiwan, and China had the highest number of developed NP-CDSSs, which may, in part, be attributed to regulatory drivers existing in these countries. For example, the Brazilian Federal Council of Nursing has mandated NP implementation across all healthcare institutions [39], while Taiwan’s Health Authorities and the National Union of Nurses’ Associations have implemented similar reforms to promote health information technology (HIT) adoption [25, 94]. Additionally, support from leadership, direct involvement of ward managers, and access to technical support [53, 61, 62] are also critical factors influencing the successful implementation of NP-CDSSs, as identified in the qualitative finding of the included studies.

Finally, although NP-CDSSs are designed to support nurses’ decision-making and should absolutely not replace their clinical judgment and critical thinking, qualitative findings from this scoping review raise concerns that these systems might narrow and fix nurses’ perspectives on patient care [62, 67]. Some studies [53, 62, 67] even suggested that nurses could become passive users, merely following automated recommendations rather than actively engaging in critical analysis, potentially hindering their clinical expertise and creativity. To mitigate this risk, two studies [53, 62] recommended that NP-CDSS training should emphasize the importance of exercising clinical judgment before accepting system-generated suggestions. Additionally, nurses should be encouraged to take responsibility for identifying and reporting any suspected errors in NP-CDSS recommendations [77], a process that should be supported and streamlined by the system’s design.

### Reducing documentation burden

The documentation burden is one of the most significant challenges in today’s clinical nursing workflows, which exacerbates the strain on healthcare professionals as nursing workloads continue to rise [95]. Several technical limitations contribute to this issue, as highlighted in the qualitative findings of this review. A major concern regarding NP-CDSS implementation is the increased charting time, which reduces bedside care time, leading the nurses to perceive the system as an added burden [67]. Another key issue is the documentation of care plans, where recording non-implemented care plans increases cognitive load and overall workload [67]. Indeed, under time constraints, non-urgent planned interventions are often not prioritized and left unimplemented, even though their planning still contributes to nurses’ workload. To address these challenges, the ICS4NP-CDSS framework highlights essential features aimed at reducing documentation burden and cognitive overload. These key features include meaningful alerts — such as automatic consistency checks between entries and missing data notifications, while also acknowledging the risk of alert fatigue, a well-documented issue that can reduce clinical attention to important warnings [96]. Additionally, intelligent integration of NP with other hospital information system modules, including laboratory results, radiology findings, medical diagnoses and notes, and measurement instrument outcomes, is essential [61, 62].

Another important feature is the automatic data retrieval or pre-filling at the assessment step to reduce manual data entry. However, only four systems (19%) [36, 56, 71, 75] included this feature. Additionally, interoperability with external databases, including medical (24%) (5/21), laboratory (9.5%) (2/21), and evaluation scales (24%) (5/21) (such as pain, fall, and pressure ulcer scales), remains limited. The lack of interoperability can hinder the assessment step and consequently the ND step. Although voice recognition is not yet a standard feature in ICS4NP-CDSSs, it is an emerging technology that, when combined with the aforementioned functionalities, could significantly reduce the documentation burden [97].

Another critical usability factor is ergonomic system design. However, only eight systems (38%) [54–58, 60, 66, 69, 72–75, 77, 78, 80, 82, 87–89] explicitly addressed ergonomic considerations. Additionally, qualitative findings indicate that complex navigation across multiple screens and poor integration with interdisciplinary interfaces are major barriers to the use of NP-CDSSs [57, 61, 62, 74]. Addressing these limitations is essential to enhance usability and support the successful implementation of NP-CDSSs.

All the systems retrieved in this review used knowledge-based inference engines, but only one system integrated non-symbolic artificial intelligence (AI) techniques, such as machine learning [71]. AI holds significant potential in nursing practice, as demonstrated across various healthcare domains including nursing [98–105]. In the context of NP-CDSSs, AI can automate problem identification based on patient record data, streamline nursing process tasks such as data extraction from clinical narratives through natural language processing (NLP) and generative AI, support voice recognition and text generation to reduce manual documentation time, thereby freeing clinicians from time consuming administrative tasks [106]. Despite the promising potential of AI technologies, further research is needed to confirm their utility in nursing practices.

Only one system included a nursing workload measurement feature, utilizing the Fugulin scale to determine team sizing based on patient dependency levels [58]. This feature is crucial for nursing management, as it enables workload evaluation to optimize staffing distribution, implement safe staffing practices, and improve quality of nursing care, particularly in the context of nursing shortages. Although the Nursing Intervention Classification (NIC) incorporates workload measurement, this feature is currently not integrated into NP-CDSSs using NIC [107]. Incorporating workload assessment tools in NP-CDSSs could provide nursing managers with a valuable resource for ensuring equitable patient assignments, staffing optimization, and improved nursing care quality.

### Ensuring compliance with ICS4NP-CDSSs

This review highlights a gap in the incorporation of ICS4NP-CDSS best-practice recommendations for the design and implementation of NP-CDSSs, particularly in achieving comprehensive coverage of all NP steps and establishing clear linkages between them. Notably, 17 out of the 21 systems (81%) were developed after the publication of the ICS4NP-CDSS standard, underscoring the importance of evaluating the extent to which this standard has been adopted. In line with previous studies [23, 24], our findings show that only seven (36%) of the 21 systems included in this review covered all NP steps, with implementation and outcome evaluation steps being the least implemented. Although outcome evaluation is a crucial step, ensuring the completion of the care plan or prompting reassessment, it may not routinely be performed in daily practice. Moreover, one system merged the outcome evaluation step with a new assessment phase, effectively re-initiating the NP cycle [67].

Regarding the linkages between NP steps, our review found that most NP-CDSSs (81%) were able to suggest NDs when nursing assessment components were entered, and that 71% offered automatic linkages from diagnosis to intervention selection. However, while nearly half of the systems (48%) provided automated linkages between NDs or interventions and the formulation of expected patient outcomes, only 24% included outcome criteria related to NDs at the evaluation step. These findings align with Virginia Saba’s assertion [108] that decision support rules facilitating ND recommendations based on assessment data are well established in the literature. However, the final step, outcome evaluation, requires further enhancement [108]. It is important to recognize that some SNLs have already built automatic linkages between NDs, interventions, and patient outcomes through their taxonomies [26, 31, 32, 36].

Another critical feature for a comprehensive NP-CDSS is the ability to enter free text, allowing flexibility in documenting complex patient cases. However, only three systems included this option [39, 53, 59, 63–65].

Furthermore, qualitative findings from nurses revealed an additional practical need not adequately addressed by current NP-CDSSs or ICS4NP-CDSS recommendations: the lack of a prioritization function to help nurses rank NDs and interventions, enabling them to create realistic and feasible care plans. Zhai et al. emphasized that this functionality is crucial [53, 62], and its integration into future NP-CDSSs—and potentially into future ICS4NP-CDSS versions—should be explored. While this may be a complex challenge, it is essential for enhancing the practical usability of NP-CDSSs in clinical settings.

### Limitations

While this study rigorously adhered to the scoping review methodology, it has several notable limitations. Scoping reviews are particularly well-suited for mapping key concepts within a research field, identifying the main types of available evidence, and highlighting gaps in the literature. However, a key limitation of this approach is the absence of formal quality appraisal of the retrieved studies. As allowed by the scoping review methodology (Arksey & O’Malley [43]), we did not assess the quality of the included studies, e.g., we did not consider study size or design of the retrieved studies, and only considered NP-CDSS-related information. Consequently, all retrieved studies were treated at the same level, regardless of their strength of evidence—a critical limitation, as only a few studies actually assessed the impact of NP-CDSSs on outcomes.

We restricted our search to PubMed, which is known to index over 93.4% of publications [47]. Additionally, a preparatory study conducted by the authors [48] identified PubMed as the most effective bibliographic database for retrieving original articles on Nursing CDSSs. However, while PubMed is considered the most relevant database for this study, we cannot rule out the possibility that this choice led to the omission of relevant publications.

While studies pertaining to settings outside the hospital could represent topics that play an important role in improving patient outcomes and care efficiency, these studies were excluded to align with the study’s purpose of focusing on findings from research on CDSSs targeting the decisions registered nurses make in hospital settings.

## Conclusions

This scoping review highlights that the field of NP-CDSSs is still in its early stages, with widespread implementation yet to occur despite the potential of NP-CDSSs to enhance the quality of nursing care. Notably, more studies including the implementation outcomes of such systems are needed, particularly to assess the accuracy of nursing diagnoses, improvements in nursing process implementation, and reductions in cognitive burden. Although the ICS4NP-CDSS framework proposed by Müller-Staub and colleagues [29] provides best-practice recommendations for the design and implementation of NP-CDSSs, this review highlights a gap in the incorporation of ICS4NP-CDSS best-practice recommendations for the design and implementation of NP-CDSSs. While these guidelines align with the usability challenges identified in the reviewed studies, their principles were found to be only partially implemented in the examined systems.

Providing continuous decision support to NP from the initial patient assessment stage to outcome evaluation may require strengthening and expanding automatic linkages across all steps of NP in NP-CDSSs. This would help reduce the additional documentation burden associated with data entry, selection, modification, as well as the management of complex clinical concepts. To enhance the perceived usefulness of NP-CDSSs, these systems should support nursing clinical reasoning while also integrating non-nursing data (e.g., medical and laboratory results) automatically. Additionally, automating certain aspects of nursing data entry and workflow could further alleviate documentation and cognitive burden. All these findings underscore the need for more usability studies on NP-CDSSs to better identify facilitators and mitigate barriers to their effective implementation.

## Electronic supplementary material

Below is the link to the electronic supplementary material.

Supplementary Material 1

## Data Availability

Data is provided within the manuscript or supplementary information files.

## Abbreviations

AI: Artificial Intelligence
CCC: Clinical Care Classification
CDSS: Clinical Decision Support Systems
COVID-19: Coronavirus Disease of 2019
ICNP: International Classification for Nursing Practice
ICS4NP-CDSSs: Internationally Consented Standard for NP-CDSSs
NANDA-I: International Classification of Nursing Diagnoses
ND: Nursing Diagnosis
N-EHR: Nursing Electronic Health Records
NIC: Nursing Interventions Classification
NMDS: Nursing Minimum Data Set
NOC: Nursing Outcomes Classification
NP: Nursing Process
NP-CDSS: Nursing Process Clinical Decision Support Systems
PICO: Problem, Intervention, Comparison, Outcomes
PRESS: Peer Review of Electronic Search Strategies
PRISMA-ScR: Preferred Reporting Items for Systematic Reviews and Meta-Analyses extension for Scoping Reviews
SNL: Standardized Nursing Languages
SNOMED-CT: Systematized Nomenclature of Medicine-Clinical Terms
