# Supplementary material for: Capabilities of computerized decision support systems supporting the nursing process in hospital settings: a scoping review
Source: BMC Nurs. 2025 Jul 1;24:677. doi: 10.1186/s12912-025-03272-w (PMC12210972; doi:10.1186/s12912-025-03272-w)
Supplement: Supplementary file 1 — Supplementary Material 1 [file 12912_2025_3272_MOESM1_ESM.docx]

**Supplementary material 1: PUBMED QUERY**

2013:2024[DP]

AND

"journal article" [PT]

AND

hasabstract

AND

("decision support"[TIAB] OR "decision making" [TIAB] OR "guidance" [TIAB] OR "guideline-based" [TIAB] OR "practice guideline*" [TIAB] OR "clinical guideline*" [TIAB] OR "clinical pathway*" [TIAB] OR "computer-assisted decision*" [TIAB] OR "computer-aided decision*" [TIAB] OR "computer-supported decision*" [TIAB] OR "experts system*" [TIAB] OR "reminder system*" [TIAB] OR "clinical reminder*" [TIAB] OR "computer reminder*" [TIAB] OR "electronic reminder*" [TIAB] OR "computerized reminder*" [TIAB] OR "computerised reminder*" [TIAB] OR "alert system*" [TIAB] OR "computer alert*" [TIAB] OR "electronic alert*" [TIAB] OR "computerized alert*" [TIAB] OR "computerised alert*" [TIAB] OR Decision Support Systems, Clinical [MH] OR Decision Support Systems, Management [MH] OR Decision Making, Computer-Assisted [MH] OR Expert Systems[MH] OR Clinical Alarms [MH] OR Reminder Systems [MH])

AND

("computer*"[TIAB] OR "software*"[TIAB] OR "electronic health record*"[TIAB] OR "electronic patient record*"[TIAB] OR "decision support system*"[TIAB] OR "information technolog*"[TIAB])

AND

("nursing"[TIAB] OR "nurse"[TIAB] OR "nurses"[TIAB] OR Nursing [MH] OR Nurses [MH] OR Nursing Staff [MH] OR Nursing Care [MH] OR Nursing Process [MH])

AND

("hospital" [TIAB] OR "hospitals" [TIAB] OR "hospitalis*" [TIAB] OR "hospitaliz*" [TIAB] OR "medical unit*" [TIAB] OR "clinical unit*" [TIAB] OR "medical center*" [TIAB] OR "clinical setting*" [TIAB] OR "ward*" [TIAB] OR "department*" [TIAB] OR "resident" [TIAB] OR "residents" [TIAB] OR "electronic health record*" [TIAB] OR "electronic patient record*" [TIAB] OR information system*" [TIAB] OR "intensive care" [TIAB] OR "acute care" [TIAB] OR "emergency" [TIAB] OR "emergencies" [TIAB] OR Hospitals [MH] OR Hospital Units [MH] OR Hospital Departments [MH])
